# 

**Published:** 1859-08

**Authors:** 


					﻿PUBLISHED MONTHLY, AT S3 PER ANNUM IN ADVANCE.
A.TL A.ZN'T.A
MEDICAL WO SHI
I 0 I 1 ■ 1 £ ®
EDITED BY
JOSEPH P. LOGAN, M. D.,
Professor of Physiology and Diseases of Women and Children in the Atlanta Medical
College.	.
AND
W. F. WESTMORELAND, M. D.,
PROFES30R OF THE PRINCIPLES AND PRACTICE OF SURGERY IN THE ATLANTA MEDICAL COLLEGE,
Pax et scientia, sed veritas sine timore.
/ >
Vol. IV. AUGUST, 1.859. 7 J^XIL
ATLANTA, GEORGIA:
PRINTED BY J. I- MILLER & CO.
(Successors to G. P. Eddy & Co.)
1 8 59.
EACH NUMBER CONTAINS SIXTY-FOUR PAGES.
I
c
c
s
5
fi
I
?
ft
ft
ft
®
z
ft
©
??
X
ft
©
ft
>
ft
ft
ft
ft
>>
ft
•n
a
ft
□
VT
T
—
s
©
©
ft
ft
a
I
a
X
ft
-a
5
a
X
ft
ft
X
a
ft
ft
o
>fi
ft
X
ft
ft
ft
ft
ft"
ft
a
5.
ft’
5
X
ft
ft*
ft
ft
ft
ft
a
S
>
X*
ft
X
ft
a
©
3
ft
ft
X
				

